# Report of Diseases in the Public and Private Practice of One of the Physicians of the Finsbury Dispensary, from the 20th of January to the 20th of February

**Published:** 1806-03

**Authors:** J. Reid

**Affiliations:** Grenville Street, Brunswick Square


*Report of Diseases in the public and private Practice of one of the Physicians of the Finsbury Dispensary, from the 20th of January to the 20th of February.*

| | | | |
|---|---|---|---|
| Dyspepsia | 11 | Podagra | 1 |
| Asthenia Senilis | 2 | Epilepsia | 3 |
| Hypochondriasis | 5 | Hepatitis Chronica | 6 |
| Chlorosis | 11 | Pneumatosis | 4 |
| Menorrhagia | 3 | Nephritis calculosa | 2 |
| Fluor Albus | 5 | Hæmorrhois | 1 |
| Hæmoptysis | 1 | Dysenteria | 2 |
| Phthisis Pulmonalis | 3 | Diarrhœa | 5 |
| Peripneumonia Notha | 5 | Rubeola | 8 |
| Hydrops Pectoris | 1 | Morbi Infantiles | 17 |
| Rheumatismus | 7 | Morbi Cutanei | 15 |

In the above list no individual disease appears particularly prominent. There has of late been no epidemic. One of the cases of dyspepsia noticed in the catalogue might be accounted for from the decayed condition of the teeth, by which they were rendered unequal to the due performance of their appropriate office.

When it is considered how much health and life itself depend upon the perfect assimilation or *incorporation* of the food—that such an assimilation must be preceded by an adequate digestion.—and that this latter process cannot be well effected without a previous and sufficient mastication, cation, the function of the teeth will appear to approach in dignity and consequence to that of the most important viscera. The wanton and unnecessary extraction of a tooth ought to be avoided, not on account merely of the momentary pain of the operation, or of the appearance of dilapidation which it gives to the face, but because it involves the loss of one of the instruments most essentially and intimately connected with the preservation of vigour and the continuance of vitality.

By medical inquirers who have taken pains to search into this particular subject, it has been remarked, that one of the circumstances that have almost invariably attended among the phenomena of old-age, is the unimpaired integrity of the teeth. Their decay, which for the most part accompanies, cannot fail likewise to contribute to and accelerate that gradual reduction of substance and of strength which so uniformly characterizes the more advanced stages of our existence.

It is of high moment that parents and others connected with the superintendance of persons in very early life, should sufficiently attend to and feel the importance of these remarks.

It ought not to be overlooked, that while the state of the teeth produces an effect on the stomach, the diseased condition of the latter is not unfrequently communicated to the former.

One of the cases of typhus fever alluded to in the catalogue is alarming, and will not improbably prove fatal, in consequence of being complicated with a consumptive affection. The corroborating and stimulating remedies necessary for the one, being directy calculated to aggravate the symptoms and increase the danger of the other.*

A person has recently been attended by the writer of this article, who died of a fairly exhausted excitability. It is remarkable that his wife, who had been in that relation for nearly fifty years, survived her intimate associate only a few weeks, without any apparent or assignable cause

---

* The Parisian practice, which the Reporter had an opportunity, some years ago, of observing at the Hotel-Dieu, in febrile complaints, would not interfere with an inflammatory or tubercular affection of the lungs. It consisted principallly of bleeding, ptisans, and *du vin*, which last was in fact wine only to the *eye*. It was weaker than our small-beer, and was singularly well calculated to induce that degree of diarrhœa which ought to be particularly guarded against in fevers of extreme debility.

cause of disease, except the disruption of so long an union, and amalgamation, as it were, of minds.

The vital principle of two individuals may, from congeniality or habit, be so identified or combined, that it would be impossible to destroy the one without fatally injuring the other. It is somewhat like that sympathy by which if the right eye, in consequence of weakness or disease, become blind, the left seldom retains long afterwards its capacity of vision.

Several instances of disease have very recently occurred, which arose evidently from want of occupation. "Employment is every thing."* For disorders characterized by languor, the radical remedy is exertion. Leisure, in its physical operation, is not seldom almost equally injurious with licentiousness. Business attended with extreme care and uneasiness, is perhaps more desirable than the having no subject at all of uneasiness or care. An atmosphere, however contaminated, proves less certainly and immediately destructive than an absolute vacuum. In the laborious and luxurious extremes of society, the same effect may be observed to originate from causes apparently opposite.

The former, by a total deprivation of hope, the latter by possessing so much as to have nothing left to hope for, are too apt to take refuge in debauchery or excess. In either case a similar calamity eventually ensues. If indiscretion of this kind merely curtailed life, it might admit of some apology, or even of vindication; but a forced hilarity, or an artificially induced stupefaction, does not, like the guillotine, separate at once the slender thread of vitality. For the most part it produces this effect through the medium of a slow and agonizing disease. A laborious and protracted interval of dropsy, schirrous liver, palsy, or some other equally fearful disorder, in most instances occurs between the loss of a capacity for enjoying life, and the period of its ultimate and entire extinction.

Death is the last, but not the worst, result of intemperance

J. REID.

*Grenville Street, Brunswick Square, Feb.* 24, 1806.

---

* Paley.

